# Family Interaction and Consensus with IT Support

**DOI:** 10.1155/2012/269637

**Published:** 2012-12-03

**Authors:** Peter Karlsudd

**Affiliations:** School of Education, Psychology and Sport Science, Linnaeus University, 391 82 Kalmar, Sweden

## Abstract

Experience shows that there are great defects in information and collaboration between families and professionals in the health and care sector. In an attempt to improve the quality of the efforts planned and implemented in collaboration with relatives a family-related IT-based collaboration system called CIDC was constructed. With the intention to facilitate communication, information, documentation, and collaboration the system was tested together with parents of children with cognitive impairment. The system contains a number of functions gathered in a so-called e-collaboration room. The person administering and distributing the system authorizes the patient/care recipient or relative to build up an e-collaboration room. The result has been largely positive, but the part, which was supposed to document everyday activities, leaves much to be desired. For this reason a follow-up study was completed, and an iPad was used as a contact book, which with the help of the Dropbox software provided increased insight into the child and improved the contact with parents without losing confidentiality or causing extra workload for the staff. By automatic download from the iPad parents and/or contact persons could easily follow the documentation of children's everyday activities.

## 1. Introduction

In health, long-term care, and rehabilitation and habilitation efforts there are often several different professions involved. Each one of these actors makes important decisions and carries out activities of importance to the decisions and efforts made by other participants. A familiar problem in these processes (chains of care) is that the communication does not work as desired. Frequently decisions are made of which families and professionals should partake but the information does not reach them quickly and effectively enough. Quite often there is a lack of coordinated planning and a joint approach, even though this is prescribed in policy documents at all levels [1, 2]. Sometimes families and the different professional groups do not know who are included in activities around a person that is subjected to, for example, rehabilitating efforts, let alone what overarching methods apply in the efforts. At other times the various measures work in opposite directions, which is inefficient, and in the worst case creates problems and suffering. For people who take part in two or three activities every day the contact may be bad between, for instance, a residence for the intellectually disabled and a school or workplace (daily activity).

Not seldom does it happen that confidentiality between different organizations and professions obstructs collaboration. Nor is it unusual that relatives are denied the right conditions for getting informed about and committing themselves to the coordinated efforts. Since different efforts of rehabilitation and habilitation depend on each other, it is essential that all information is correct, up-to-date, and coordinated and that everybody concerned obtains the right channels for sharing and contributing to information that may benefit life circumstances and development [3, 4]. In order to improve the previous action on the basis of the CIDC system and develop communication between health professionals and family further experiments have been made. The result of this work is described in this paper.

### 1.1. Confidentiality, Integrity, and Profession

Confidentiality and personal integrity are two concepts of great importance in the health and care sector. Misinterpreting these concepts may lead to erecting barriers, resulting in activities in which families are marginalized. To plead confidentiality and refuse to give out information to those directly involved may often violate the principle of the individual good. With regard to functionally impaired children, for example, consideration must be taken to both short- and long-term consequences of their physical and mental wellbeing and development. Due to a wrong interpretation of the confidentiality act such a child may be regarded as “even more different and deviant” from others, in other words, as being so special that it can only be dealt with by some carefully chosen persons who protect it from an indifferent environment. Nor is it unusual that the confidentiality concept is invoked to mark professional status between one profession and another.

Personal integrity is a loadstar in health and care, but the concept may be used by staff to evade commitment, empathy, and activity. If residents sit inactive and alone in their rooms, it is not always a matter of their own will, and it should not be possible to refer to this or to arguments like “protecting individual integrity.” With a better activity documentation, as well as an increased insight into the experiences of those next of kin, discussions of whether certain activities are suitable from the perspective of self-determination principles or not may form the basis of what decisions to make.

Allowing professionals to work in a protected milieu without a good contact with families may foster a specific professional lingo and a negative jargon by which these experts, albeit unwittingly, tend to establish a distance to their environment. The consequence will be that the distance between caregivers, care recipients, and families increases. Families may feel excluded by the fear of making errors and the feeling of lacking sufficient knowledge about commitment and responsibility. Offering more insight and collaboration may counteract the outsider feeling experienced by families. Since there exists a given and valuable role for close relatives to play in health and care [5, 6], the idea of a family-related IT-based collaboration system has developed, which was presented in its initial form in 2008 [7]. This paper describes the experience that generated a follow-up development as documented in the form of case studies.

### 1.2. Introducing New Technology

It is becoming increasingly common to search for more efficient solutions in the health and care sector by means of modern information technology [8, 9]. The role of IT support will become even more prominent in future health care. A great many people use the computer and the Internet frequently to search for information on health care issues [10]. Several studies show that the quality of these services may become at least as good as traditional meetings [11, 12]. According to Dahlbom and Mathiassen [13] there are three different categories controlling the development of IT-based organization systems. The first strategy aims at automatizing work, which means that there are no fundamental changes in the organization. According to the second strategy, the IT system keeps developing for improving or solving problems at work, which leads to organizational changes. The third strategy is used for creating a new organization altogether, in other words, a total and radical transformation. In the work described in this paper I would, on the basis of the division above, define the planning as an attempt to improve and solve problems in the organization.

Introducing a new service or system often involves motivational as well as practical problems. To introduce IT-based tools into an organization is not altogether easy but is often a complicated time-consuming process. Communication systems are based on strong interactivity between users and computers. The “soft” human part of the system is generally the most important factor for success. Using digital technology in professional communication is more of a paradigm shift involving changes of work culture than a question of purely technical solutions [14, 15].

There are a number of obstacles that make people oppose changes. These may be divided into practical, psychological, value, and power barriers [16, 17]. If a thoroughgoing transformation is to take place, these barriers must be overcome. Practical obstacles may comprise the economy, availability, educational opportunity, lack of time, and so forth. One psychological barrier may arise because professionals feel threatened in their roles, perhaps coupled to fears that the care recipient's relatives may know more. Value barriers may entail that the values that come with the technology do not agree with those of the professional. When a power barrier emerges, the entire professional role may be challenged.

### 1.3. Digital Possibilities for Family Collaboration

Everyday family life has become increasingly digital and mobile. Privately utilized technology with computers, iPads, and mobile phones can also solve a number of issues within health and care. This wealth of access to social media may facilitate communication between those involved in the health and care sector. Text-based communication surfaces like Facebook, Twitter, blogs, and e-mail lists, as well as visual and sound communication like Skype and FaceTime, are available for free. In addition, these services are web based and do not require any special installations. With such resources there is no excuse for avoiding the most fundamental element in health and care, namely, documentation and communication.

The disadvantages of well-known free web applications may be the feeling that the collaboration networks people create are not protected against insight from unauthorized persons. Among other disadvantages may be that they are linked to advertising, which gives a nonserious impression, that there are more or fewer functions than desired, or that it becomes difficult for users to distinguish their private from their professional role. One advantage of using a specially adapted application is that the necessary functions are gathered in one place and that the negative debate which might be associated with the more familiar communication tools will not affect the attitude towards utilizing this one.

Naturally, the contact taking place in personal meetings face to face in physical space is the most important, but this does not rule out contacts via digital media. These can offer excellent tools for building up important contact surfaces with families and for improving healthcare. It is of the utmost importance to keep the aim of involving families, not the technology, in focus.

### 1.4. The E-Collaboration Room

In order to focus more on families a web-based system for health and care was constructed, called CIDC, which is an abbreviation of the variables the system is expected to improve: communication, information, documentation, and collaboration. The system was constructed at Linnaeus University. The basic construction consists of a number of functions gathered in a website referred to in the system as the e-collaboration room (e-room). A fairly experienced computer user can quickly set up an e-room and invite collaboration members with the support of a number of easily adaptable and useable functions. The functions include quick messages via a website, SMS, or e-mail. They also comprise a calendar for coordinated planning and evaluation, a search function for structured documentation, a discussion forum, a diary, an individual presentation of the organization, and a picture diary, as well as file processing and a presentation of patient journals and weblinks [7].

The chief administrator responsible for the system authorizes the user/patient or relative to take on e-room responsibility, thus giving access to organizing and administering an e-collaboration room. The latter then invites members to the room and decides which functions and rights each participant should have access to.

Usually the care recipient is the e-collaboration room administrator, or it may be the parent of a functionally impaired child, but it can also be a professional like a nurse, perhaps an assigned registered nurse, or a contact person. This person decides on user rights and on which system modules should be accessible. The confidentiality between the different professional groups is waived by the care recipient or guardian in order to promote a more efficient care, rehabilitation, or habilitation. What is most important is that the user has a large influence over the system and thus becomes more motivated for the efforts that are planned [1, 18].

All participants can easily introduce themselves and their organization on their own system homepage by filling in a text and posting a picture. This enables each organization/individual to visualize important documents like minutes, measures planned, and links relevant to the activity.

On the message page everyone can pass on information by sending a text and attachments. The message will reach the recipient/s as an e-mail or SMS, and the addressees can be limited to one or two persons.

The calendar constitutes the basis of the system. By double-clicking a box in a planning calendar it is possible to post an activity, to which an evaluation can be added later. One can also attach documents, pictures, and films of activities and evaluations. The calendar offers a good overview of when, what, how, and why an activity is planned. In the search function one can search and list information via eligible search words. This function is most useful when measures and efforts are to be evaluated, summarized, and revised [7].

## 2. Method

The method used in system development, implementation, and data collection can be defined as qualitative. The process is reminiscent of the attitudes in action research, which strives to increase critical thinking and not to shy away from new problems [19–21]. The action research approach has been far from unambiguously formulated, different researchers emphasizing different aspects. However, a central concept is the close link between theory and practice [19, 20]. Different terms have been used in an attempt to better define the approach. Argyris et al. [22] uses the term action science for the part of action research which is more theoretically oriented and action research for the practically oriented part. Regardless of the emphasis, the obvious common denominator of action research is researching through action. This project required the participants to work with pedagogical development from a clear perspective of change. There is a distinct difference between the roles of course participants and researcher in the action research process. Tiller [23] developed this circumstance and describes the role of the former as action learning, whereas action research puts the emphasis on what the latter does. The researcher's task is to study and analyze how the action research method has changed, while the task of the course participants may be to describe and reflect on their development work [21]. This study is divided into two sections, of which the first can be described as systems development and action research, while the second has a more case-based approach.

### 2.1. Data Collection

#### 2.1.1. Study 1

In the first study eight parent representatives in families with mentally disabled children were offered to use and evaluate the CIDC collaboration system. Every parent receiving the invitation has been positive to this. Six of these parents have been alone responsible for the administration of their e-room. All respondents participating in the study were contacted by the author via e-mail with the result that 47 out of 62 possible users in the eight e-rooms (76%) chose to take part. Three users declined participating and 12 users never answered, which gives an external dropout of 24%. The majority of those declining participation have probably not been active in the system or have been negative to its use. The questionnaire distributed consisted of 20 questions, where the respondent could express the system's pros and cons as well as the degree of utilization and the value of the system functions. The questions were presented four months after the e-rooms had been implemented. Half of the e-rooms centred on in the study were introduced on an occasion when every participant had an access to a computer and was able to test and ask questions on the system. The other four were introduced via e-mail with a manual attachment. The questions were estimated on a five-grade scale, where 1 represented a very low/negative estimate and 5 a very high one. The questions to the users were constructed on the basis of the goals (questions 1–5) formulated for the system [7].

#### 2.1.2. Study 2

Since it was shown in the first study that the daily everyday documentation via text and pictures in the calendar and picture diary functions was not satisfactorily utilized, another development process started. To facilitate and increase students' or residents' possibilities to control their own documentation, a mobile digital contact book, in the form of an iPad, was introduced in addition to the CIDC program. The second and follow-up phase of the study carried out can be defined as a case study where three families with a handicapped child were placed in nursing home. The number of respondents who participated in this study was 6 parents and 15 employees. All were interviewed about their experiences.

## 3. Results

### 3.1. Study 1

The result of the study shows that the majority of the participants were positive to using the system. The most positive were those parents who, together with their children, were focused on in applying the system and who only saw advantages with coordinating the communication. According to them, coordination, continuity, and quality had improved considerably. The function that was appreciated the most was the message function which, in their view, processed correct information quickly to everyone involved.

The message function was also highly appreciated by other actors. The planning function was liked and used by some parents and staff, while documentation, evaluation, and search were the functions used the least. From interviews and comments it can be gleaned that the task of evaluating was felt to be time consuming and strenuous by the employees involved.

Quite a few gave expression to the advantages arising when the parent waives the confidentiality to the benefit of an open exchange of information. This increases the knowledge and understanding of the efforts made. One of the users called it “pure continuing education.” The result shows that from a user-technical perspective the system can be easily implemented and that the communication between the actors increases considerably. Parents gain an increased insight, overview, motivation, and knowledge with regard to the efforts made. Most of the users thought that the information was presented in a well-structured manner and found the system user-friendly. Some of the professional users expressed anxiety that the system might involve an increase rather than effectivization of the workload [7].

Another highly positive experience from this study was that parents could waive confidentiality between the different actors and thus contribute to making important information accessible to those affected by the efforts. It is the user that forms the starting point of the system, deciding who may participate and what functions should be accessible. By in-depth interviews with the staff it appeared that there was a fair amount of opposition to documenting work using a camera and then writing and posting pictures on a usually stationary computer.

### 3.2. Study 2

The experience of the extended development process demonstrates that when the diary takes a physical shape and accompanies the student as a natural tool between, for example, school and residence, the documentation is much more thorough-going, especially with regard to sound messages, films, and pictures. It also turns out that students, unless they are hampered by a complex handicap, become more active in their documentation.

Using an iPad has a great many advantages, the foremost being the built-in camera and microphone enabling people to easily document their everyday life in different locations. Besides, the mobile web allows people to communicate by text, sound, and pictures through various applications without being tied to a specific environment. In the study the iPad came to function as a physical contact diary accompanying the student/care recipient all day, and facilitating for the staff in different parts of the organization to view pictures and films and discuss the day's events with the student/care recipient without having to find a computer. One problem which derived from this solution was, however, that parents or guardians were unable to read and follow everyday activities, because they could perhaps only take part of the iPad information every second weekend when they met the student. To solve this problem the student's iPad was connected to a service called dropbox, which enables selected programs to automatically download information onto a server to which other users are given access and authorization. In the study referred to here pictures and texts in programs used in the digital contact book could be copied for easy access to parents, without the information being transmitted by the staff. This setup enables several users, via protected codes, to obtain the information without having physical access to the iPad. The text editor used for this purpose was Plain Text, a free application.

## 4. Discussion

In some of the e-collaboration rooms activity was low among some of the participants in the study. The reason may have been that they had a negative attitude to the system, perhaps due to fear of an increase in the workload, a lack of IT competence or displeasure at making the activity more transparent.

Reverting to Dalin's [16] barriers to IT implementation, nearly all the power barriers were represented in the study. There are practical obstacles in the form of insufficient training and equipment. A psychological barrier is the increased insight into and comparison between organizations. In a few cases a value barrier is apparent when someone has expressed anxiety about the openness inherent in the system. None of the respondents stated that their tasks would be threatened, while a few felt being called in question because of the increasing insight into and demands on the organization (a power barrier).

In those e-collaboration rooms where a parent had been responsible the assessment was that the usefulness of functions and the extent of utilization were higher than in e-rooms where someone else was responsible for the system. A reasonable assumption is that a parent is often better at (or more persistent in) getting those around to feel responsible for making information and knowledge transfer more effective. In order to achieve a higher degree of utilization it has been found that a physical introductory meeting to launch the system is to be preferred.

As for the confidentiality requirement there is no difference in using the iPad. Codes can be used for accessing the iPad and its applications both physically and via the web. Besides, if the iPad is stolen, all information can be erased by accessing the web.

One strength in providing documentation by an Internet-connected iPad is that it is physical as well as asynchronous and synchronous. The iPad offers a flexible mobile utilization of a series of communication programs. Being regarded in many environments as a natural tool for both work and leisure increasingly enables information to be produced and received by everyone involved. Staff feeling unfamiliar with new technology may express anxiety about the iPad being sensitive and expensive as an excuse for not using it. Such notions and arguments must be dedramatized, as iPads are both relatively inexpensive and very durable.

To calculate profits in economic terms in the above study is difficult. Still, it is evident that the quality of the interaction between care recipients, families, and caregivers has risen. There are quite a few respondents who believe that the quality of information and documentation has become considerably better and easier to achieve.

This is a very positive outcome, as it enables families to participate in, for example, the long-term care of residents and to discuss suitable methods and approaches with the staff involved. Greater insight and influence are key words and well-founded requirements for every health and care activity. In long-term care with the focus on the family digital tools may offer great help towards attaining these aims.

## References

[1] Åhgren B (2001). Chains of care: a counterbalance to fragmented health care. *Journal of Integrated Care Pathways*.

[2] Karlsudd P (2007). The “narrow” and the “wide” activity: the circumstances of integration. *The International Journal of Disability, Community & Rehabilitation*.

[3] Acker GM (1999). The impact of clients’ mental illness on social workers’ job satisfaction and burnout. *Health and Social Work*.

[4] Lloyd C, King R, Chenoweth L (2002). Social work, stress and burnout: a review. *Journal of Mental Health*.

[5] Levin B, Normann R (2000). *Opportunity of Care*.

[6] Coulter A (1999). Paternalism or partnership?. *British Medical Journal*.

[7] Karlsudd P (2008). E-collaboration for children with functional disabilities. *Telemedicine and e-Health*.

[8] Harun MH (2001). Integrating e-Learning into the workplace. *Internet and Higher Education*.

[9] Zhao Y, Yagi Y, Nakajima I, Juzoji H (2002). IP telephony—new horizon for telemedicine and e-health. *Journal of Medical Systems*.

[10] Lu C, Wirrell EC, Blackman M (2005). Where do families of children with epilepsy obtain their information?. *Journal of Child Neurology*.

[11] Peterman TW (2000). Elements of success at a traditional/virtual university: lessons learned from three years of growth in cyberspace. *Journal of Academic Librarianship*.

[12] Berger NS (1999). Pioneering experiences in distance learning: lessons learned. *Journal of Management Education*.

[13] Dahlbom B, Mathiassen L (1993). *Computers in Context—the Philosophy and Practice of System Design*.

[14] Jandér K (2005). *Access to Digital Learning Resources in Higher Education—A Pilot Study*.

[15] Karlsudd P (2010). For benefit or oblivion? From idea and vision to the implementation and support of learning applications. *International Journal of Advanced Research in Computer Science*.

[16] Dalin P (1978). *Limits to Educational Research*.

[17] Karlsudd P (2011). *Support for Learning—Possibilities and Obstacles in Learning Applications*.

[18] Wold-Olsen P (2002). The customer revolution: the pharmaceutical industry and direct communication to patients and the public. *Eurohealth*.

[19] Feldman A, Minstrell J, Kelly AE, Lesh RA (2000). Action research as a research methodology for the study of the teaching and learning of science. *Research Design in Mathematics and Science Education*.

[20] Archer JM, Holly ML, Kasten WC (2001). *Action Research for Teachers*.

[21] Karlsudd P, Tågerud Y (2008). Bridging the gap—taking the distance out of e-learning. *The Electronic Journal of e-Learning*.

[22] Argyris C, Putnam R, McLain Smith D (1985). *Action Science*.

[23] Tiller T, Svein (1995). Action learning and action research—opportunities and dilemmas. *Reflections on Educational Research*.

